# Deep learning based image reconstruction algorithm for limited-angle translational computed tomography

**DOI:** 10.1371/journal.pone.0226963

**Published:** 2020-01-06

**Authors:** Jiaxi Wang, Jun Liang, Jingye Cheng, Yumeng Guo, Li Zeng

**Affiliations:** 1 Key Laboratory of Optoelectronic Technology and System of the Education Ministry of China, Chongqing University, Chongqing, China; 2 Engineering Research Center of Industrial Computed Tomography Nondestructive Testing of the Education Ministry of China, Chongqing University, Chongqing, China; 3 College of Computer Science, Civil Aviation Flight University of China, Guanghan Sichuan, China; 4 College of Mathematics and Statistics, Chongqing University, Chongqing, China; 5 College of Mathematics and Statistics, Chongqing Technology and Business University, Chongqing, China; Zhejiang University, CHINA

## Abstract

As a low-end computed tomography (CT) system, translational CT (TCT) is in urgent demand in developing countries. Under some circumstances, in order to reduce the scan time, decrease the X-ray radiation or scan long objects, furthermore, to avoid the inconsistency of the detector for the large angle scanning, we use the limited-angle TCT scanning mode to scan an object within a limited angular range. However, this scanning mode introduces some additional noise and limited-angle artifacts that seriously degrade the imaging quality and affect the diagnosis accuracy. To reconstruct a high-quality image for the limited-angle TCT scanning mode, we develop a limited-angle TCT image reconstruction algorithm based on a U-net convolutional neural network (CNN). First, we use the SART method to the limited-angle TCT projection data, then we import the image reconstructed by SART method to a well-trained CNN which can suppress the artifacts and preserve the structures to obtain a better reconstructed image. Some simulation experiments are implemented to demonstrate the performance of the developed algorithm for the limited-angle TCT scanning mode. Compared with some state-of-the-art methods, the developed algorithm can effectively suppress the noise and the limited-angle artifacts while preserving the image structures.

## Introduction

Translational computed tomography (TCT) as a new low-end CT system, which can obtain the interior image without destroying the scanned object by using the projection data obtained from the detector, is created for developing countries [1]. It utilizes translation to realize linear scanning, where the X-ray source and the flat panel detector are placed face to face with an object between them and are moved in opposite directions during the scanning process. When the projection data collected from the TCT are complete, filtered back projection (FBP)-type algorithms can accurately reconstruct some high-quality images [2, 3]. However, in some practical TCT applications, in order to reduce the scan time, decrease the X-ray radiation which may cause potential risks to patients, or scan some long objects within a limited angular range, furthermore, to avoid the inconsistency of the detector for the large angle scanning in the translational scanning scheme, the obtained projection data of the scanned object are usually incomplete.

In this circumstance, some artifacts are presented in the image reconstructed by the FBP-type method. Algebraic reconstruction algorithms, such as the simultaneous algebraic reconstruction technique (SART) [4] and the algebraic reconstruction technique (ART) [5], have better denoising effect than FBP method when the projection data are complete. However, if the available projection data are incomplete, these methods cannot obtain satisfactory reconstructed image.

In recent years, researchers are becoming increasingly interested in regularized iterative reconstruction algorithms for incomplete projection data, as these algorithms can add some prior knowledge to obtain better reconstructed image and will not be affected by the geometrical structure of the scanning mode. Hence, more and more researchers are keen to construct an appropriate transformation that can utilize prior information of the reconstructed object, and various regularized iterative reconstruction algorithms have been proposed [6–10]. As one of the regularized iterative reconstruction algorithms, total variation (TV)-based minimization method [11] can suppress the streak artifacts and noise when the projection data are acquired within a few-views scanning mode. However, some limited-angle artifacts will appear on the edges of the object in the reconstructed image when the projection data are acquired from limited-angle CT. In addition, staircase effect or blocky artifacts will also appear in the reconstructed image due to the assumption of the reconstructed image is piecewise constant. To address this problem, Lauzier [12] proposed an image reconstruction algorithm based on the prior image obtained from previous scanning. Then, Chen [13] utilized the prior knowledge of the known actual scanning range to propose an anisotropic total variation (ATV) method for improving the reconstructed image quality from limited-angle projection data. Wang [9] proposed a limited-angle CT image reconstruction algorithm based on the wavelet frame, and the reconstructed images show that it has advantage in suppressing noise and slope arifacts. Recently, Wang [14] incorporated the reweighted technique into the ATV method, and proposed a new iteratively reweighted ATV method to solve limited-angle CT reconstruction problem. Yu [15] found that the regularization term based on the L0-norm of the image gradient can better preserve the edge of the image, and they proposed an edge-preserving image reconstruction method for limited-angle CT. In summary, these regularized iterative reconstruction algorithms can reduce the limited-angle artifacts and noise to some extent. However, it is difficult to choose the appropriate regularization terms and adjust the regularization parameters, and these choices play a decisive role in the quality of the reconstructed images.

Nowadays, deep learning [16] has emerged as a potential method providing promising performance for image classification [17] and segmentation [18]. In recent years, deep learning has also been applied to the CT image reconstruction problem [19, 20]. Pelt et al. [21] developed a convolution neural network (CNN) which can be treated as a weighted combination of the FBP method and some learned filters, the experimental results verify that it is better than directly using FBP method for the few-views CT. In [22], Boublil utilized a CNN to integrate multiple reconstructed results to get a better reconstructed image compared to other iterative reconstruction algorithms. Chen et al. [23] utilized a CNN for post-processing of a single reconstruction result from the low-dose projection data, and experimental results show that it has advantage in structure preservation and artifact reduction. Yang et al. [24] proposed a measure named as perceptual similarity to measure the loss, which can prevent the mean squared error from overly smoothing of the image. Jin et al. [25] proposed a novel deep CNN called FBPConvNet, and they demonstrated the performance of FBPConvNet in sparse-view reconstruction for parallel beam X-ray CT. First, they apply the FBP method to the sparse-view projection data. Second, they import the image reconstructed by FBP method to the CNN trained to make the image reconstructed by FBP method as close as possible to the label image. Finally, the end of the CNN provides the reconstructed image.

Inspired by the above researches, we combine the algebraic reconstruction algorithm with deep learning to improve the quality of the reconstructed image for the limited-angle TCT scanning mode. This paper mainly has the following contributions:

1. To reduce scan time, decrease X-ray radiation or scan some long objects, furthermore, to avoid the inconsistency of the detector for the large angle scanning in translational scanning scheme, we use a limited-angle TCT scanning mode which manually rotates 30° per unit time.
2. To deal with the limited-angle TCT reconstruction problem, we develop a deep learning based image reconstruction algorithm, which does not need to choose the regularization terms and adjust the regularization parameters.
3. Some simulation experiments show that the proposed algorithm has advantage in suppressing noise and limited-angle artifacts while preserving image structures.

The rest of this paper is organized as follows. In Section II, we derive the limited-angle TCT scanning mode. In Section III, we introduce the developed image reconstruction algorithm. In Section IV, we describe the experimental design and analyse the experimental results. In Section V, we give some discussion. In Section VI, we conclude this paper.

## Limited-angle TCT scanning mode

CT plays a key role in diagnostic imaging and intervention [26]. Generally speaking, the CT image reconstruction problem is actually solving the following equation:

Ax=b (1)

where CT imaging matrix *A* has *M* × *N* elements, *x* = (*x*_1_, *x*_2_, …, *x*_*N*_)^*T*^ and *b* = (*b*_1_, *b*_2_, …, *b*_*M*_)^*T*^ represent the discrete attenuation coefficients of the reconstructed image and the projection data collected from the detector. CT image reconstruction is to obtain the unknown *x* from the known imaging matrix *A* and the available projection data *b*.

**Fig 1:**
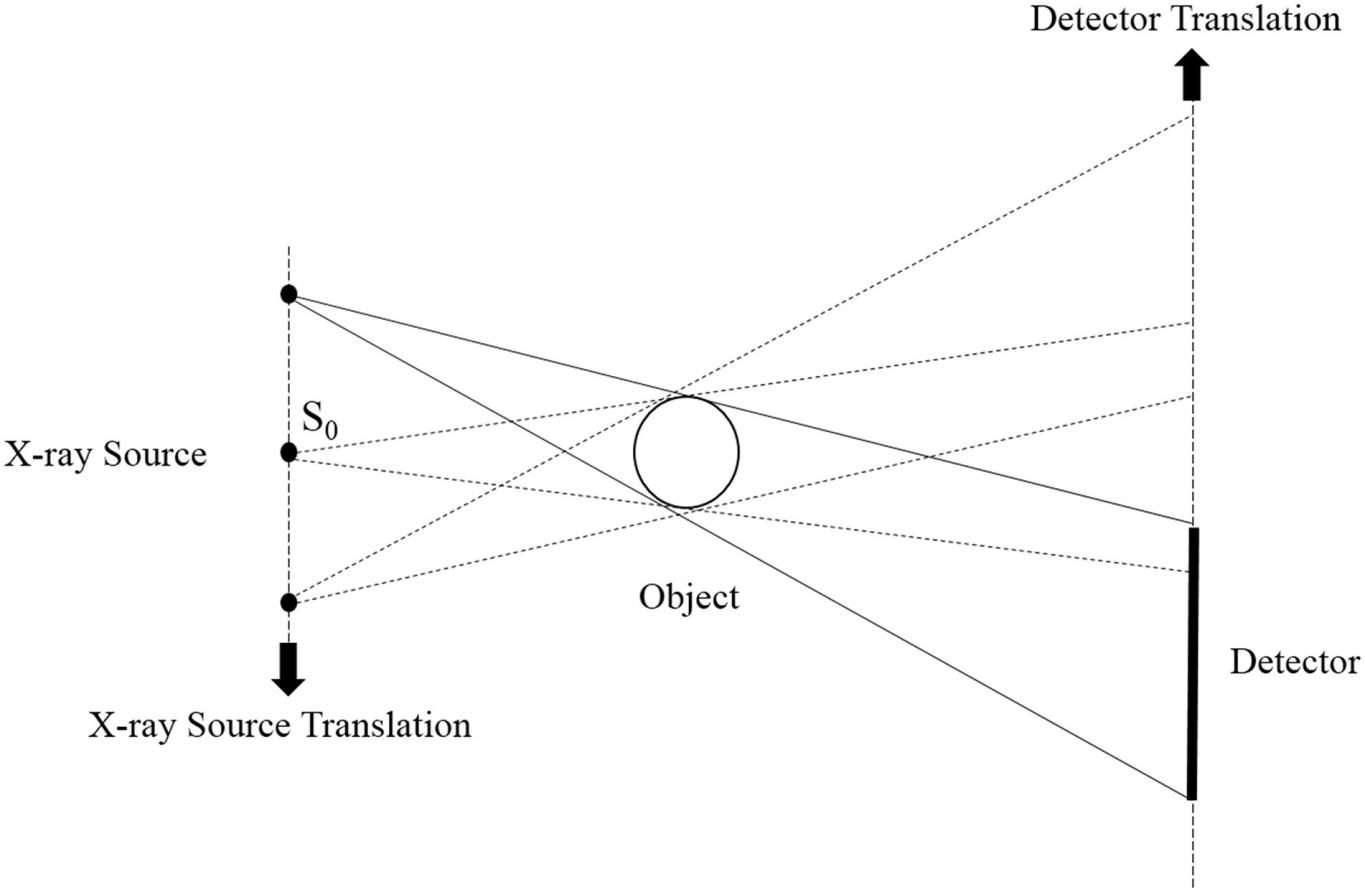
Translation based CT.

Modern CT scanners, which use sliding ring, wide-array detectors and multiple sources, have very fast rotation speed and are expensive. They are typically used by big city hospitals in developed countries and are rarely found in the rural areas of developing countries because of their high costs; therefore, a low-end CT system is required. Liu et al. [1] proposed a translation-based data acquisition mode called the translational CT system. In this system, the data acquisition scheme is based on opposite parallel linear movements. As illustrated in Fig 1, the X-ray source and the flat panel detector are positioned face to face with an object between them. During the scanning process, the X-ray source and the flat panel detector are in opposite translation and keep the object still. In other words, the gantry with an expensive slip ring is substituted by this translational technique.

**Fig 2:**
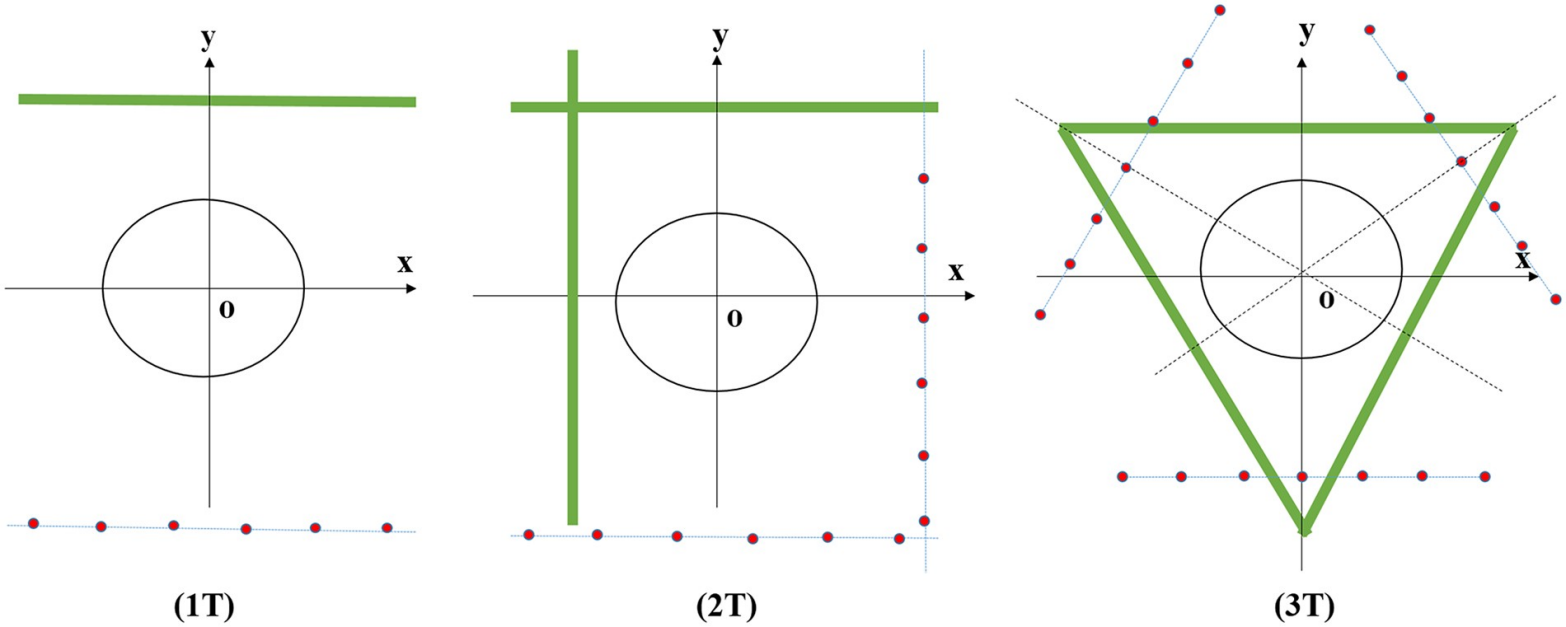
Different translational modes. The X-ray source is translated on the line where the red points located, and the corresponding flat panel detector is opposite translated on the green line.

To exactly reconstruct an object, the classic prerequisite is the complete projection data in the fan-beam geometry; i.e., the projection data should be available for an (180° + fan angle) angular range [27]. To satisfy the aforementioned classic prerequisite, the TCT projection data acquisition scheme have to rotate several times. For example, it can be manually rotated two times (2T) or three times (3T). The 1T, 2T, and 3T schemes are shown in Fig 2. The scanning process is as follows: First, we perform translational scanning and keep the object still. Second, we stop the last scanning and manually rotate the X-ray source and the detector to the next specified location, and continue to scan the object. Finally, the above operations will be repeated until the requirements are met.

**Fig 3:**
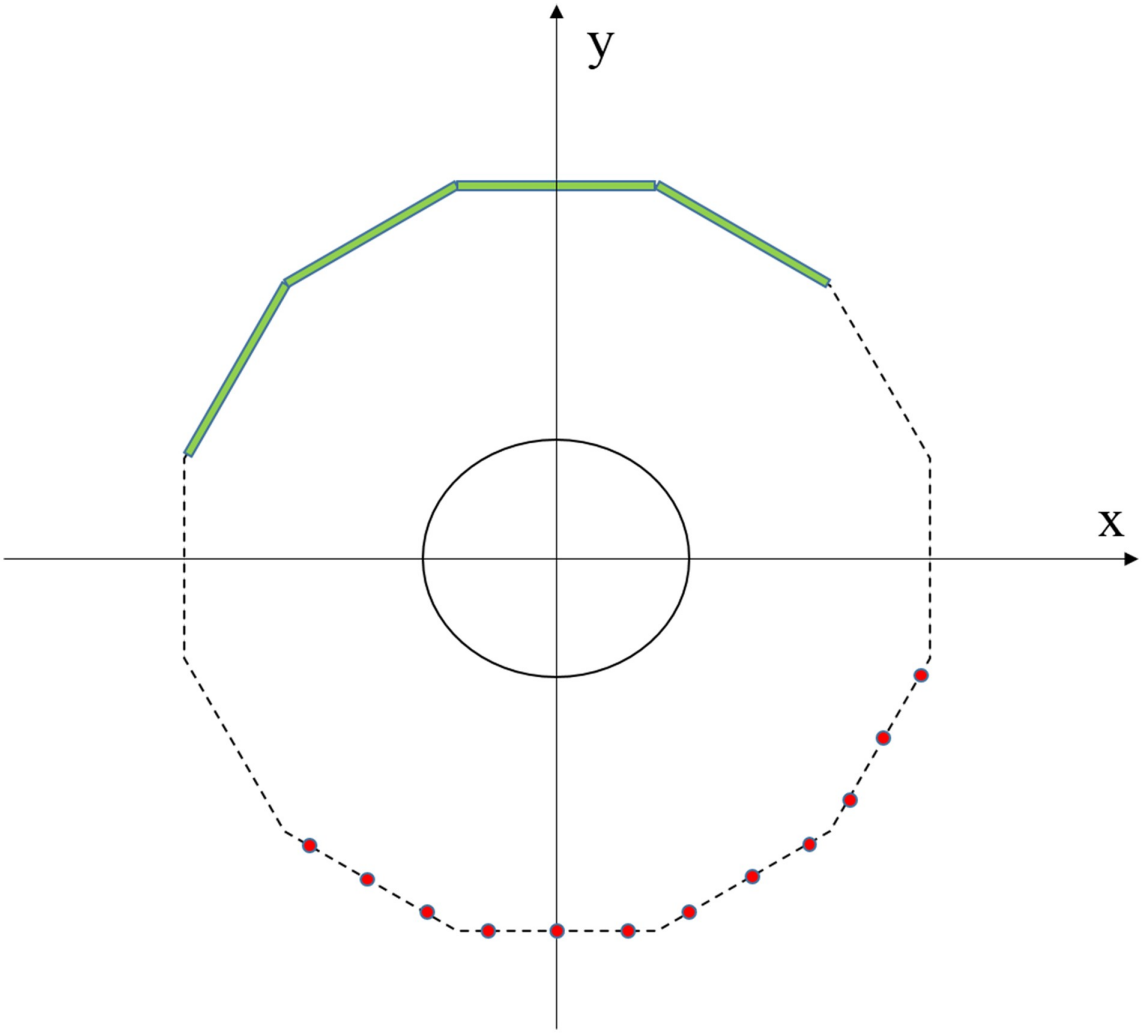
Limited-angle TCT scanning mode.

The aforementioned several projection data acquisition schemes make the flat panel detector move a long distance, and the X-ray source that is far away from the middle X-ray source S_0_ (Fig 1) needs to deflect an angle to acquire projection data because of the translational scanning scheme. However, the farther the distance is from S_0_, the greater the slant angle is needed and the worse the illumination consistency of the detector will be. To avoid the inconsistency of the detector for the large-angle scanning in the translational scanning mode, we use the smaller angle scanning mode which manually rotates 30° per unit time. Moreover, in some industry imaging applications, the X-ray source and the detector cannot be rotated many times because of the restriction of the scanning scenario, such as the wings of aircraft scanning and the pipeline in service scanning [28, 29]. In medical imaging fields, to reduce the scanning time and decrease the dose of X-ray, which may pose potential risks to patients, the patients are scanned within a limited angular range. These scenarios lead to the limited-angle TCT reconstruction problem. As illustrated in Fig 3, when the scanning angle is 30° per unit time and the scanning range is [0°, 120°], we have to rotate the detector and the X-ray source four times. This scheme requires several rotations; however, the response of the detector has better illumination consistency than the 2T and 3T scanning schemes. Since the projection data are acquired within a limited angular range, some limited-angle artifacts will present in the reconstructed image. Next, we will focus on how to solve the limited-angle TCT reconstruction problem.

## Method

In the limited-angle TCT scanning mode, image reconstruction from the limited-angle TCT projection data is an ill-posed inverse problem. Regularized iterative reconstruction algorithms, which can incorporate the prior knowledge of the reconstructed image, are usually utilized to deal with this problem. However, these algorithms are difficult to choose the appropriate regularization terms and adjust the regularization parameters. With the development of deep learning technique, Jin et al [25] proposed a post processing based image reconstruction method named as FBPConvNet, which uses FBP method to obtain the initial image for the well-trained U-net, and it demonstrated compelling results to process sparse-view reconstruction for parallel beam X-ray CT.

**Fig 4:**
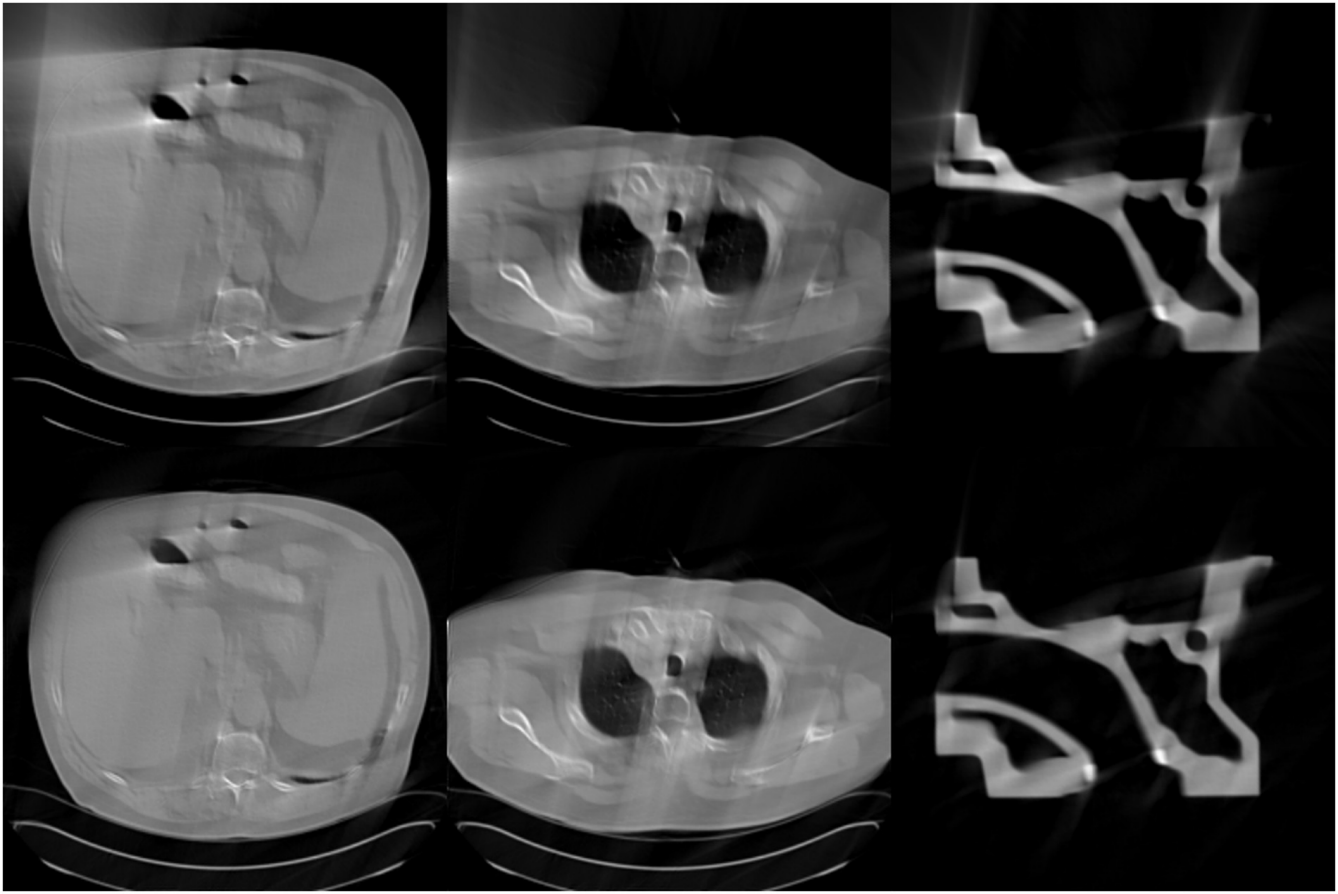
Reconstruction results for the scanning range [0°, 120°]. The first row is the images reconstructed by the FBP method, and the second row is the images reconstructed by the SART method.

Inspired by their work, we use SART method to obtain the initial image for the well-trained U-net to process limited-angle image reconstruction for TCT. The reason why we do this is that the SART method is better than the FBP method in the limited-angle TCT scanning mode (as shown in Fig 4). Therefore, the quality of the training set for the proposed algorithm is better than that of the FBPConvNet method. Moreover, in the field of deep learning, the quality of the training set plays a decisive role in the final result. Hence, the proposed algorithm which is called SARTConvNet is more effective than the FBPConvNet method for limited-angle TCT. In addition, if we use the image reconstructed by TV method as the input image of the CNN, we need to manually adjust the regularization parameters of the TV method which is a difficult job and different images have different optimal parameters.

The steps for the SARTConvNet method are as follows: First, we apply the SART method to the limited-angle TCT projection data which are obtained by the simulated experiments. Then, we import the image reconstructed by the SART method to the CNN trained to make the image reconstructed by the SART method as close as possible to the label image. Lastly, in the final layer of the CNN, a convolutional layer is used to make the CNN output a single channel image, which is the final reconstructed image of the SARTConvNet. Next, we will introduce the details of the SARTConvNet method.

### Simultaneous algebraic reconstruction technique

In the network training stage, the CNN is trained with a subset of pairs (*T*_*q*_, *L*_*q*_), where *T*_*q*_ is the image reconstructed by the SART method from limited-angle TCT projection data, *L*_*q*_ is the corresponding label image. The formula for the SART method [4] is as follows:

$$f_{j}^{\left(n+1\right)}=f_{j}^{\left(n\right)}+\beta \frac{1}{a_{+j}}\sum_{i=1}^{M}\frac{a_{ij}}{a_{i+}}(b_{i}-A_{i}f^{\left(n\right)})$$ (2)

where *n* is the number of iterations and *β* is the relaxing factor. Further, we choose *β* = 1 in this case,$a_{i+}\equiv \sum_{j=1}^{N}a_{ij}\neq 0$, *i* = 1, …, *M*, $a_{+j}\equiv \sum_{i=1}^{M}a_{ij}\neq 0$, *j* = 1, …, *N*. Here *b*_*i*_ − *A*_*i*_*f*^(*n*)^ is the difference between the actual projection data and the simulated projection data. As the iterations increase, *b*_*i*_ − *A*_*i*_*f*^(*n*)^ → 0, *f*^(*n*)^ → f*, and f* is the label image.

### Deep convolutional neural network

**Fig 5:**
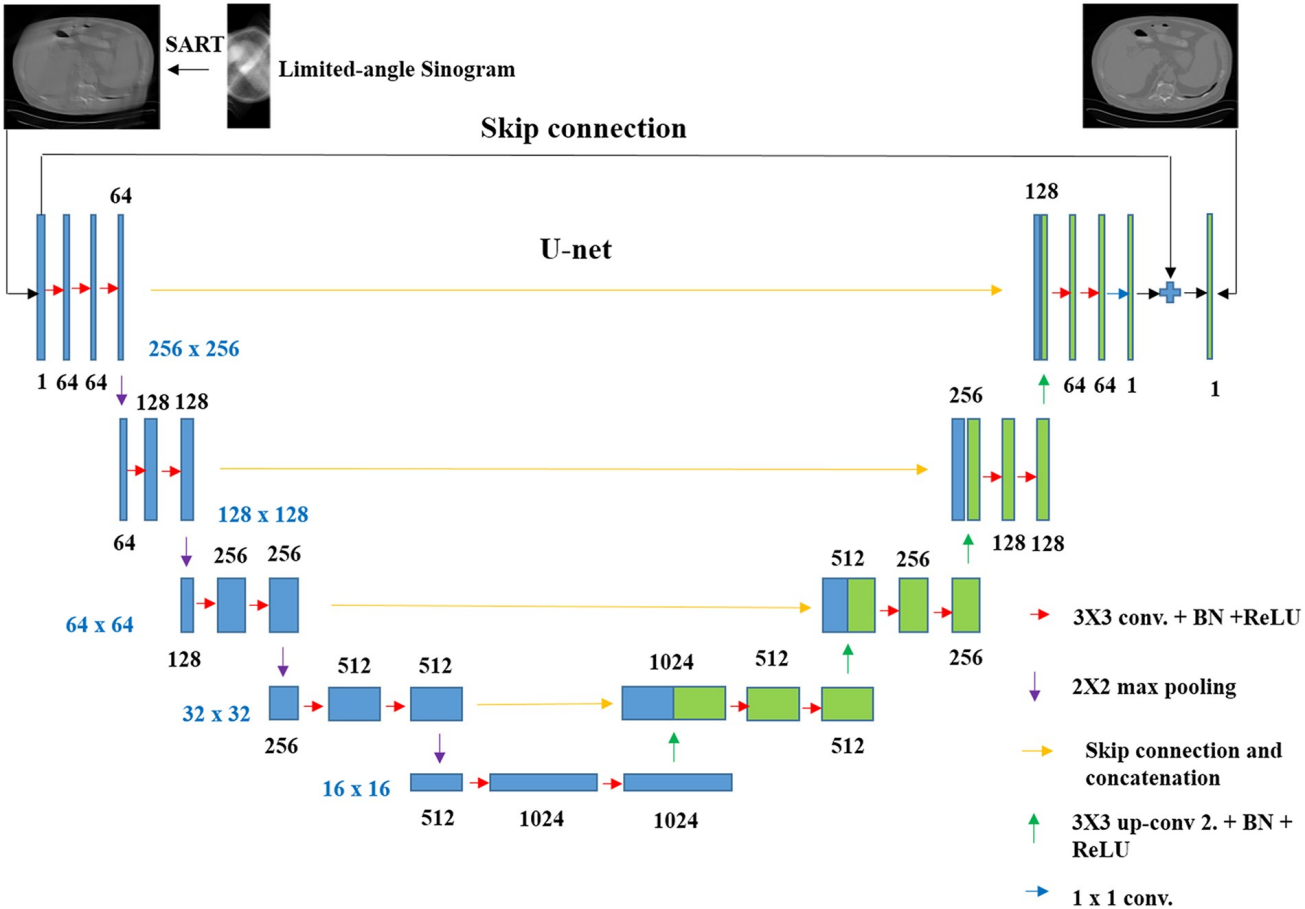
Architecture of SARTConvNet.

As shown in the Fig 5, the CNN we use in this paper is based on the U-net, which is firstly applied to image segmentation [30]. And it is composed of a downhill path and an uphill path. The downhill path consists of numerous 3 × 3 zero-padded convolutions, rectified linear units and 2 × 2 max pooling operations. After each max pooling operation, which is used for down-sampling, we double the number of feature channels for the convolution layer to obtain more feature images which can increase the feature expression ability of the network [31]. The uphill path also consists of numerous 2 × 2 up-convolutions, batch normalizations and rectified linear units. The skip connection [32, 33] and the concatenation technique are available because of the loss of useful information in every convolution and max pooling. In the final layer of the CNN, a convolutional layer is used to make the CNN output a single channel image which is the final reconstructed image.

## Experimental process and results

In this section, we use some simulation experiments to test the feasibility and effectiveness of the proposed algorithm for the limited-angle TCT scanning mode. The configuration of the computer used in the experiment is as follows: Inter(R) Core(TM) i5-6500 3.20GHz is the CPU; NVIDIA GTX 1080 with 8GB memory is the GPU. In the course of the experiment, we use the MatConvNet deep learning framework [34] and the Matlab version is R2016b. We logarithmically change the learning rate from 0.01 to 0.001, choose the batch size, patch size, momentum, number of epochs and the gradient clipping value to be 1, 256, 0.99, 151 and 10^−2^, respectively. We use the GPU for training and evaluating the CNN, and the Table 1 shows the geometrical scanning parameters of the simulated TCT system. Moreover, in order to simulate the limited-angle TCT and the scanning angle is 30° per unit time, we choose to rotate the detector and the X-ray source three, four and five times, in other words, three different scanning ranges ([0°, 90°], [0°, 120°] and [0°, 150°]) are used to validate the algorithm performance for the limited-angle TCT in this work.

**Table 1:** Geometrical scanning parameters of simulated TCT system.

Parameter	Value
Distance between the center source and detector (mm)	1300
Distance between the center source and rotation axis (mm)	900
Sampling interval between two adjacent projection views (deg)	0.145
The angle between the first source and the last source (deg)	30
Number of the source	207
Number of the detector	800
Diameter of the field of view (mm)	352.90
Size of each detector element (mm)	1
Pixel size (mm^2^)	1.38 x 1.38

In this work, we choose the peak signal-to-noise ratio (PSNR) and the structural similarity index measure (SSIM) to quantitatively evaluate the proposed algorithm. PSNR is used to estimate the difference between two images, and is defined as:

$$\text{PSNR}(\text{x},\text{y})=10\times {\text{log}}_{10}(\frac{{\left(\text{max}(\text{max}(y))\right)}^{2}}{MSE})$$

$$\text{MSE}(\text{x},\text{y})=\frac{\sum_{i,j}{\left(x_{i,j}-y_{i,j}\right)}^{2}}{N}$$

where *x* is the reconstructed image, *y* is the label image, *x*_*i*,*j*_ presents pixel value in the position (*i*, *j*), *N* denotes the total number of the pixels in the image. SSIM, which is used to measure the structural similarity between the reconstructed image and the label image, is defined as:

$$\text{SSIM}(\text{x},\text{y})=\frac{\left(2\bar{x}\bar{y}+C_{1})(2\sigma_{xy}+C_{2}\right)}{\left({\bar{x}}^{2}+{\bar{y}}^{2}+C_{1})(\sigma_{x}^{2}+\sigma_{y}^{2}+C_{2}\right)}$$

where $\bar{x}$ and $\bar{y}$ denote mean values of *x* and *y* respectively. *σ*_*x*_ and *σ*_*y*_ represent the standard deviations, and *σ*_*xy*_ is the covariance. The constants *C*_1_ and *C*_2_ are set as in [35]. A good reconstructed image should provide the highest SSIM/PSNR value.

To evaluate the performance of the proposed algorithm, we choose three algorithms to compare with the proposed algorithm. (1) L0 method, which is an edge-preserving image reconstruction method for limited-angle CT [15]. (2) ATV method, which utilizes the prior knowledge of the known actual scanning range to improve the reconstructed image quality from limited-angle projection data [36]. (3) FBPConvNet method, which is an outstanding post processing-based deep reconstruction method.

### Data preparation and experimental design

It would be nice to have some real TCT reconstructed images. However, we are in the initial research phase of the TCT, and there are not enough real projection data to train the network. In addition, most of the CT cannot provide raw projection data, and we can only obtain real CT images. So we use these real CT images to simulate the limited-angle TCT projection data to demonstrate the performance of these algorithms. Moreover, we add some Gaussian noise to the simulated projection data to evaluate the robustness of these algorithms.

The dataset we use is obtained from TCIA Collections [37]. These are 500 real full-angle CT images from many patients, and DICOM is the primary file format. Among them, there are 450 training images to train the CNN, and 50 testing images to test the performance. The locations of these images are chest and abdomen, the resolution of these images is 256 × 256.

In this paper, the main steps of the experiments are as follows: Frist, the real full-angle CT images are taken as the label images. Second, these label images are used to obtain simulated projection data for the limited-angle TCT. Finally, these four algorithms are utilized to obtain the reconstructed image.

### Network training and parameter selection

The CNN part of the SARTConvNet method and the FBPConvNet method are utilized the same training strategy as that used in [25]. And the training images are many pairs of images, which reconstructed by the SART method or the FBP method and label images. For the TCIA dataset, it needs approximately 6 h to train the CNN for 151 iterations (epochs).

For a fair comparison, the parameters of these competing methods are optimized to obtain the best results in terms of the values of the evaluation index. We do this because these parameters are usually obtained by trial and error, and how to choose these parameters is still an open question to the world. In this paper, the iteration steps of the SART method is 2500, the iteration steps of TV method is 15, and the alpha value of TV is 0.1. The parameters of the L0 method are according to reference [15], and the parameters of the ATV method are according to reference [36].

### Experimental results

**Fig 6:**
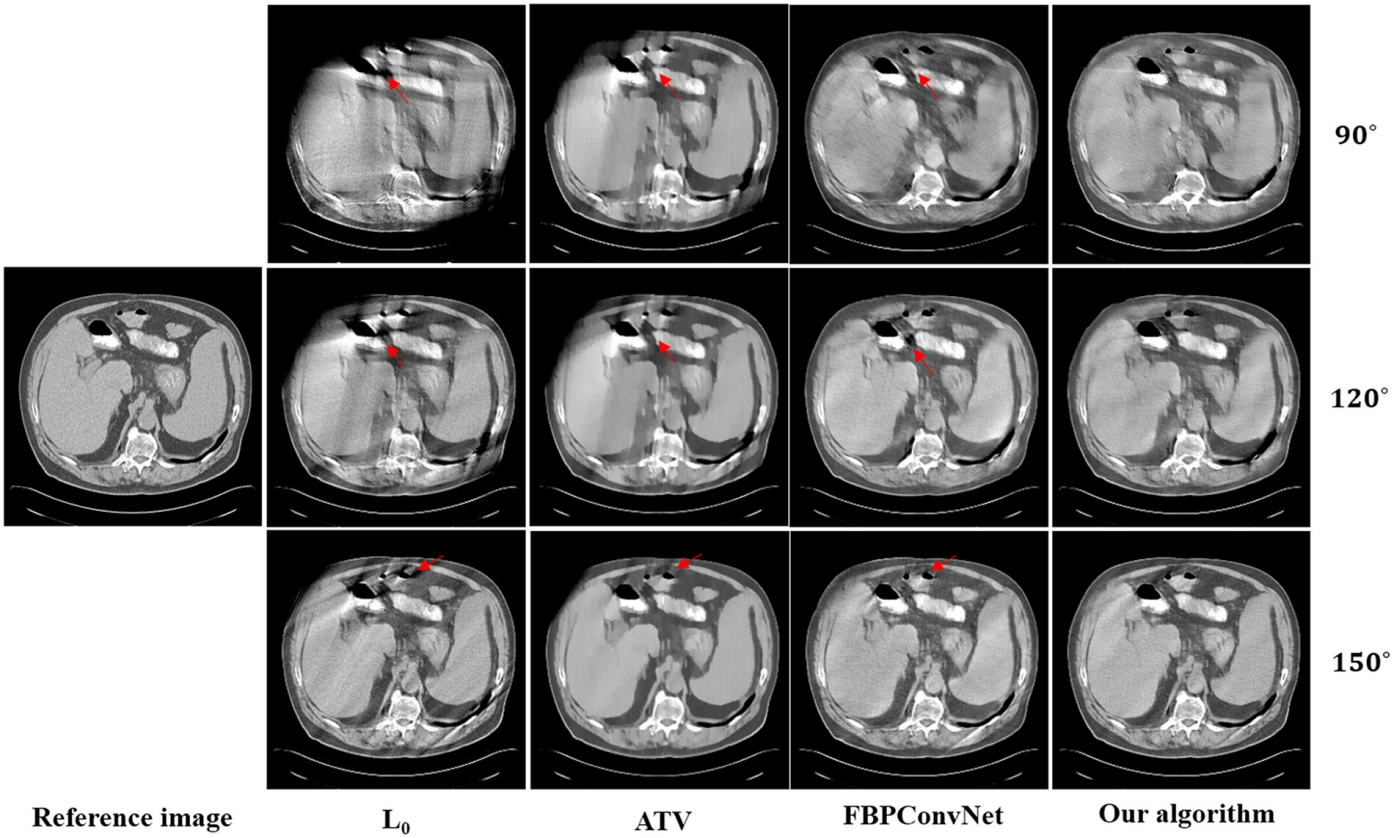
The reconstructed results of the abdomen image. The image on the first column is the reference image. The subsequent columns are the results reconstructed using L0 method, ATV method, FBPConvNet method and our algorithm. The images from top to bottom in each row are the results reconstructed for the scan ranges [0°, 90°], [0°, 120°] and [0°, 150°], respectively. The location of red arrows present some obvious artifacts, and the display window is [800 1200] HU.

**Fig 7:**
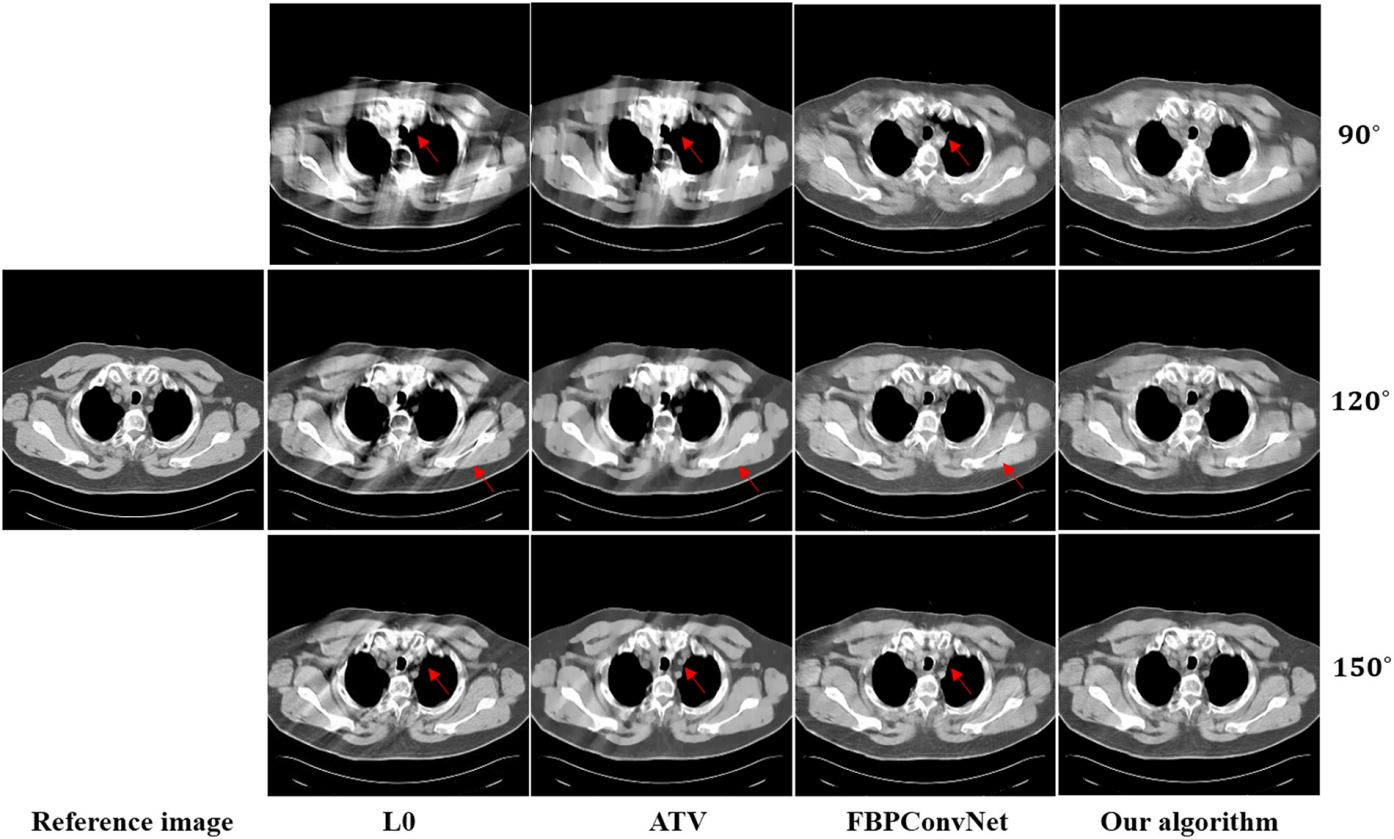
The reconstructed results of the chest image. The image on the first column is the reference image. The subsequent columns are the results reconstructed using L0 method, ATV method, FBPConvNet method and our algorithm. The images from top to bottom in each row are the results reconstructed for the scan ranges [0°, 90°], [0°, 120°] and [0°, 150°], respectively. The location of red arrows present some obvious artifacts, and the display window is [800 1200] HU.

**Fig 8:**
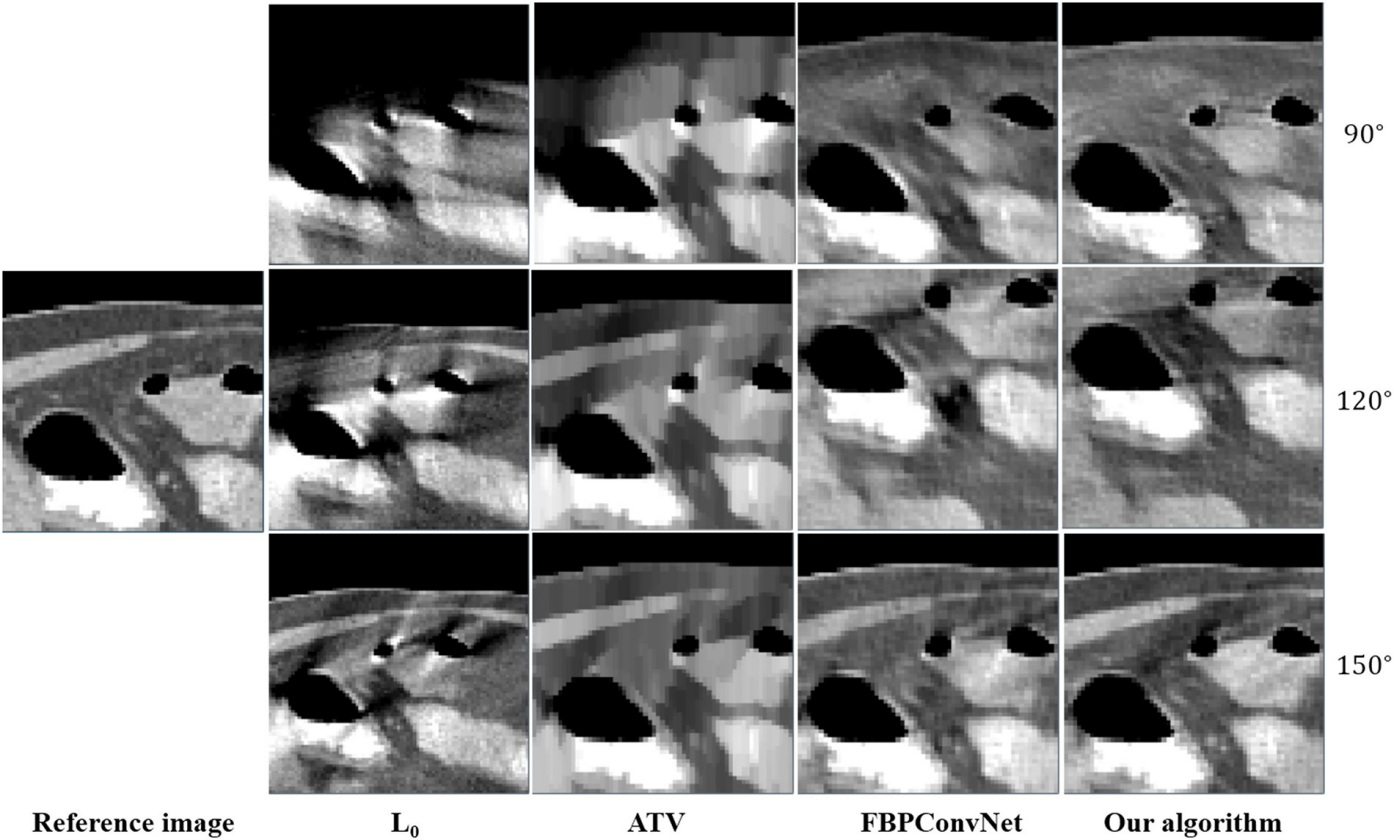
The zoom-in view of the ROIs for the Fig 6. The image on the first column is the ROI of the reference image. The subsequent columns are the ROIs of the reconstructed image for L0 method, ATV method, FBPConvNet method and our algorithm.

**Fig 9:**
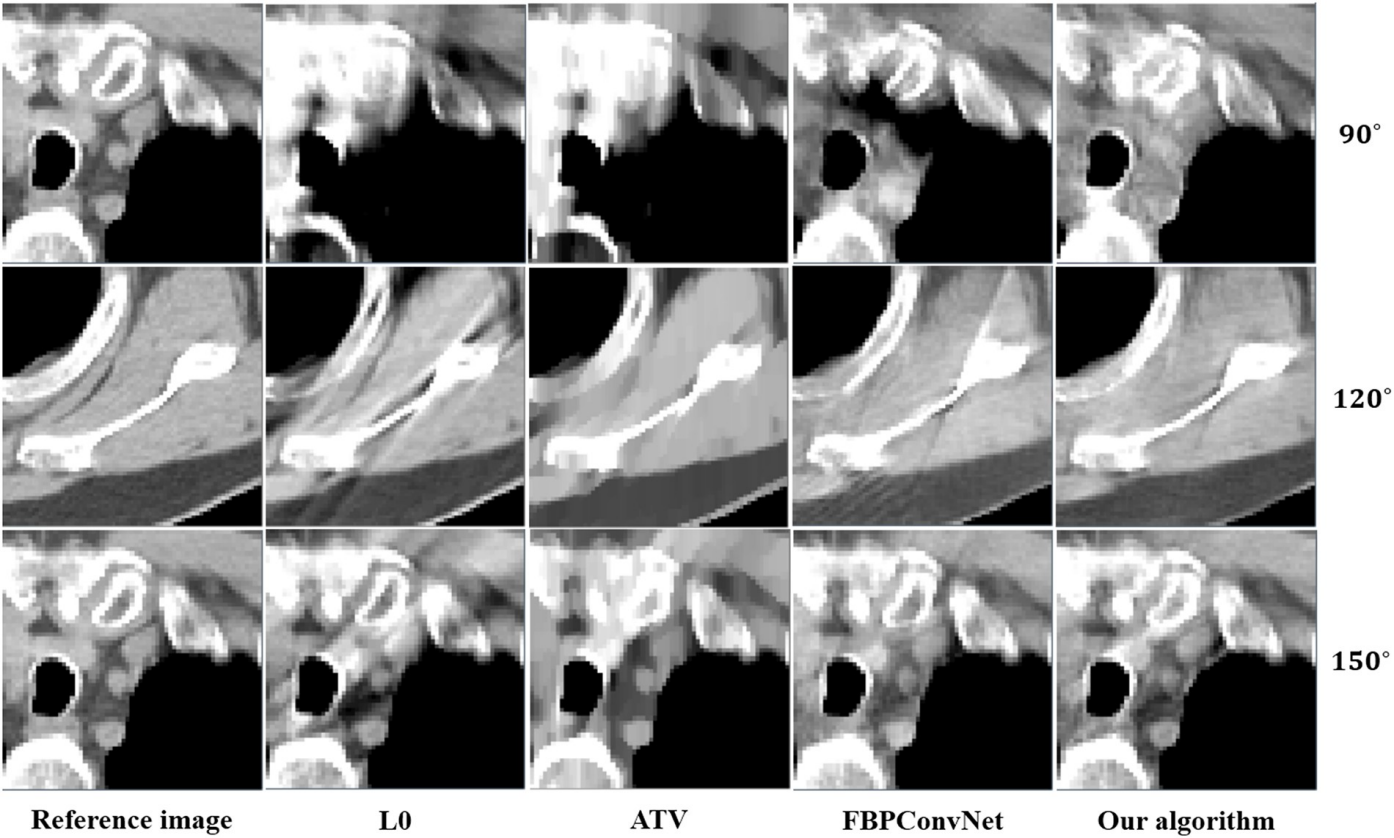
The zoom-in view of the ROIs for the Fig 7. The images on the first column are the ROIs of the reference image. The subsequent columns are the ROIs of the reconstructed image for L0 method, ATV method, FBPConvNet method and our algorithm.

In this work, we choose two representative images from the abdominal and chest regions among all of the test images to assess the performance of these four methods. Figs 6 and 7 show the reconstructed results of these two representative images. The image on the first column is the reference image. The subsequent columns are the results reconstructed using L0 method, ATV method, FBPConvNet method and our algorithm. The images from top to bottom in each row are the results reconstructed for the scan ranges [0°, 90°], [0°, 120°] and [0°, 150°], respectively. The red arrows point to some obvious artifacts which are enlarged in the Figs 8 and 9.

As seen from Figs 6–9, with the increase of the scan ranges, the quality of the reconstructed images begins to improve with different degrees. The L0 method can better preserve the edges and structures, nevertheless many limited-angle artifacts appear in the reconstructed image due to lacking projected data. ATV method can better reduce the limited-angle artifacts, however, the ATV method results in a blocky effect and smoothes some important small structures, as it assumes that the image is piecewise constant. The images reconstructed by the FBPConvNet method are better than the images reconstructed by the L0 method and ATV method. Whereas, some important details and structures have been smoothed. Our method exhibits the best performance in terms of preserving the continuous structures (such as the organ edges), suppressing the limited-angle artifacts, and retaining the inherent details (see the red arrows that indicate the region for the obvious differences).

Quantitative results associated with different algorithms for different images are listed in Tables 2 and 3. As seen from these Tables, our algorithm achieves best results for the three scanning ranges on all the indexes. Moreover, the experiments show that the larger the scanning ranges, the better the image quality.

**Table 2:** Quantitative results associated with different algorithms for the abdomen image from different angle projection data.

Scanning ranges	Algorithm	PSNR	SSIM
	L0	18.6806	0.8686
[0, 90°]	ATV	20.9951	0.8882
	FBPConvNet	24.0512	0.9007
	**SARTConvNet**	**24.9359**	**0.9043**
	L0	23.2083	0.9136
[0, 120°]	ATV	25.1692	0.9255
	FBPConvNet	26.5857	0.9276
	**SARTConvNet**	**26.8245**	**0.9467**
	L0	27.8970	0.9581
[0, 150°]	ATV	27.9213	0.9597
	FBPConvNet	28.6246	0.9416
	**SARTConvNet**	**32.8672**	**0.9782**

**Table 3:** Quantitative results associated with different algorithms for the chest image from different angle projection data.

Scanning ranges	Algorithm	PSNR	SSIM
	L0	17.4586	0.8537
[0, 90°]	ATV	18.5884	0.8811
	FBPConvNet	21.8918	0.9177
	**SARTConvNet**	**23.8079**	**0.9419**
	L0	22.2210	0.9224
[0, 120°]	ATV	23.1989	0.9328
	FBPConvNet	25.1959	0.9501
	**SARTConvNet**	**25.7111**	**0.9607**
	L0	27.1260	0.9629
[0, 150°]	ATV	28.0624	0.9689
	FBPConvNet	28.1614	0.9588
	**SARTConvNet**	**30.5538**	**0.9825**

**Fig 10:**
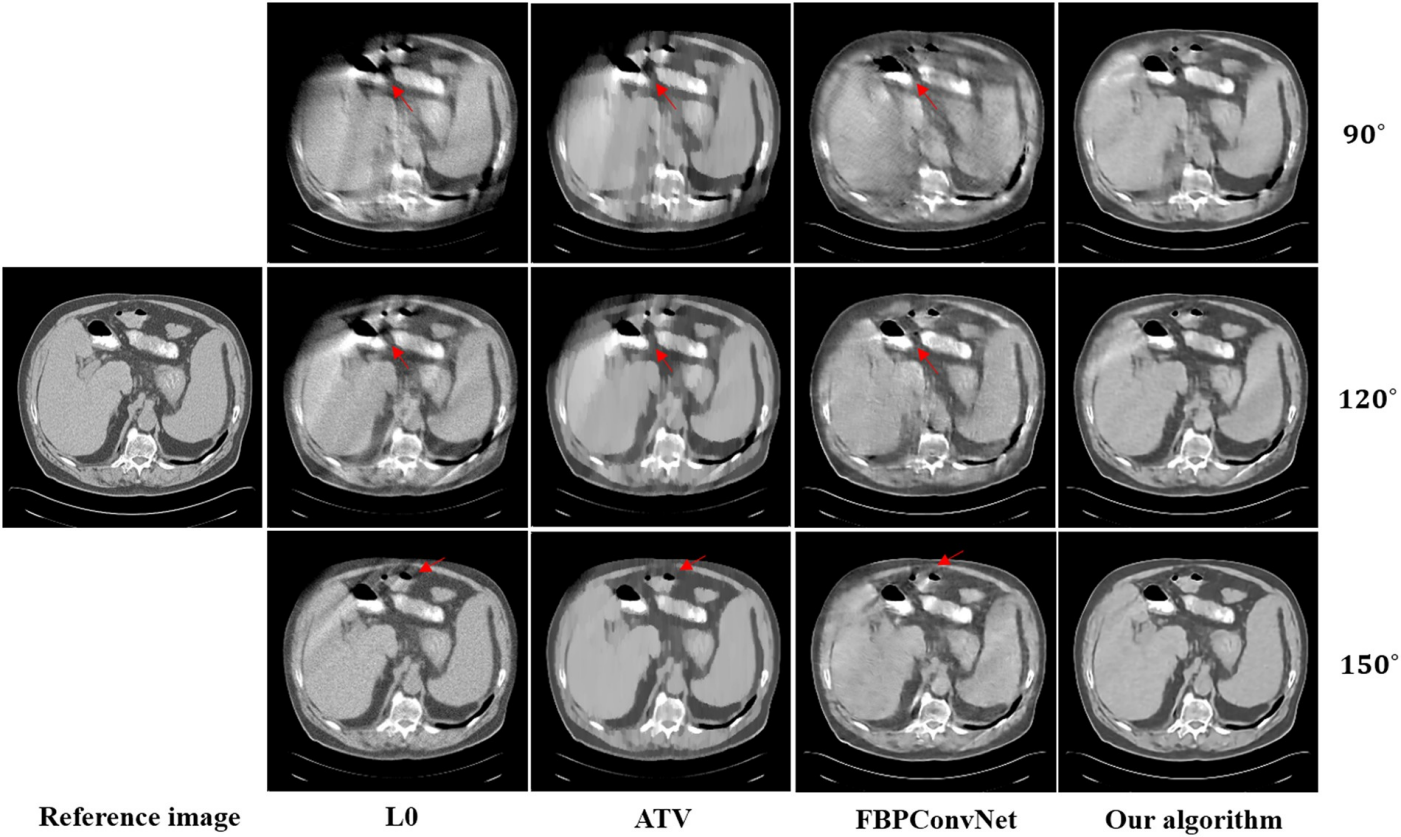
The reconstructed results of the abdomen image from the noise-add experiment. The image on the first column is the reference image. The subsequent columns are the results reconstructed using L0 method, ATV method, FBPConvNet method and our algorithm. The images from top to bottom in each row are the results reconstructed for the scan ranges [0°, 90°], [0°, 120°] and [0°, 150°], respectively. The location of red arrows present some obvious artifacts, and the display window is [800 1200] HU.

**Fig 11:**
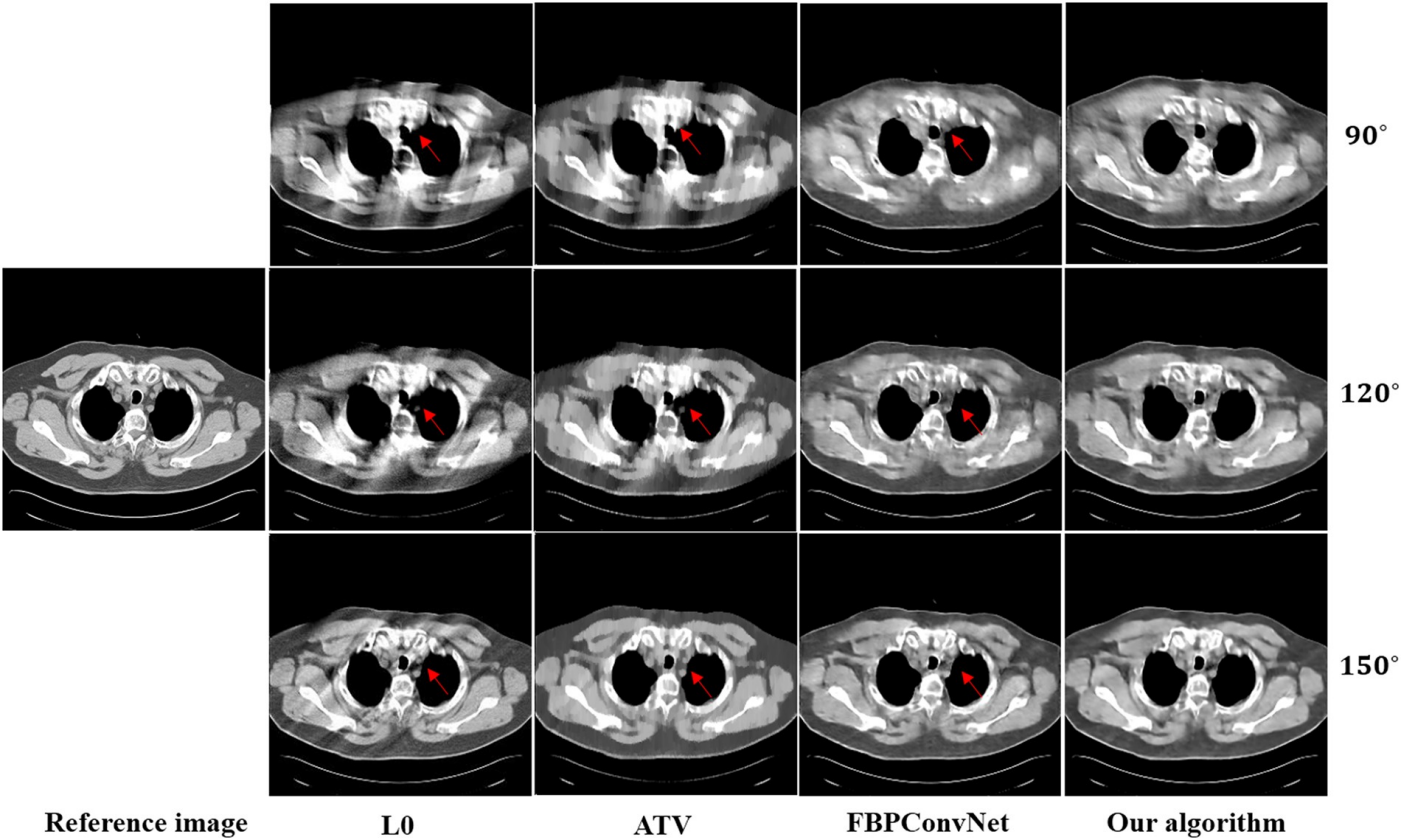
The reconstructed results of the chest image from the noise-add experiment. The image on the first column is the reference image. The subsequent columns are the results reconstructed using L0 method, ATV method, FBPConvNet method and our algorithm. The images from top to bottom in each row are the results reconstructed for the scan ranges [0°, 90°], [0°, 120°] and [0°, 150°], respectively. The location of red arrows present some obvious artifacts, and the display window is [800 1200] HU.

**Fig 12:**
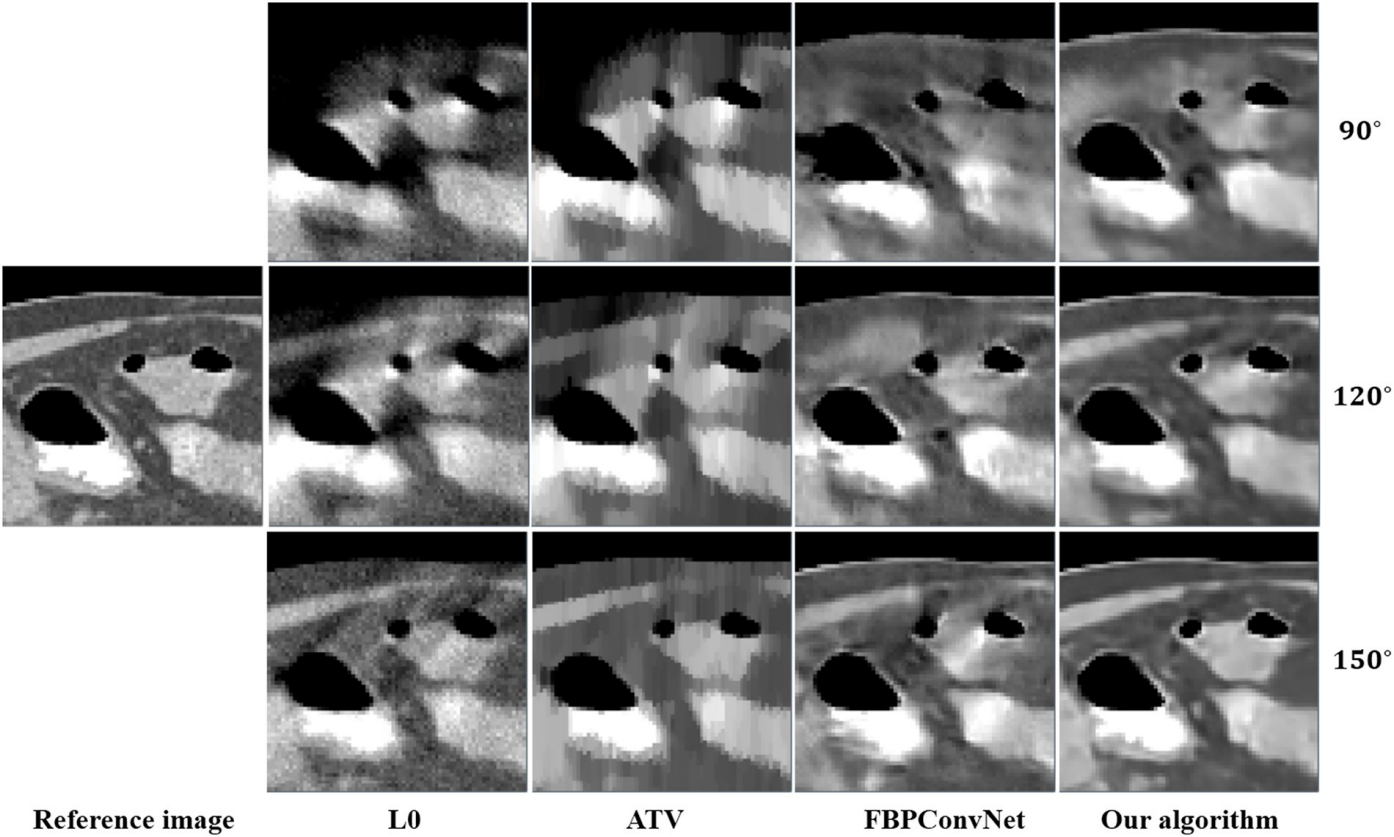
The zoom-in view of the ROIs for the Fig 10. The image on the first column is the ROI of the reference image. The subsequent columns are the ROIs of the reconstructed image for L0 method, ATV method, FBPConvNet method and our algorithm.

**Fig 13:**
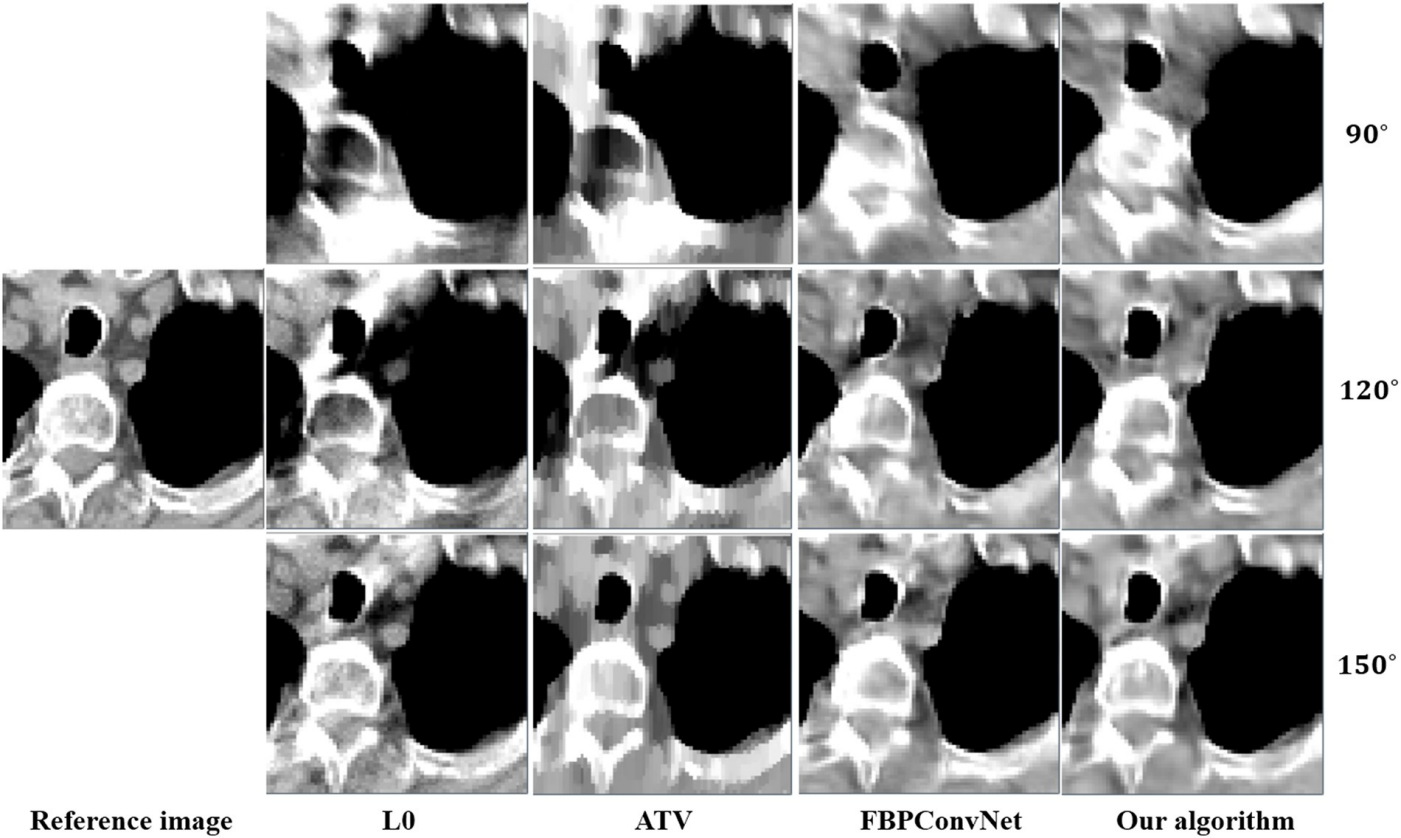
The zoom-in view of the ROIs for the Fig 11. The image on the first column is ROI of the reference image. The subsequent columns are the ROIs of the reconstructed image for L0 method, ATV method, FBPConvNet method and our algorithm.

Next, the robustness capability of these four algorithms are tested. We add the Gaussian noise (*m*, *σ*^2^) to the projection data [38], the average value m is set to zero, and the variance *σ*^2^ = 10. Figs 10 and 11 show the results from the noise-add experiment, the corresponding region of interest (ROI) results are shown in Figs 12 and 13. Besides, Tables 4 and 5 are the quantitative results associated with different algorithms for the noise-add experiment. It can be observed that the images reconstructed by our algorithm are much better than the results of L0 method, ATV method and FBPConvNet method. Compared to other three methods, our algorithm can better reduce the limited-angle artifacts, suppress the noise and preserve the continuous structures.

**Table 4:** Quantitative results associated with different algorithms for the abdomen image from different angle noise-add projection data.

Scanning ranges	Algorithm	PSNR	SSIM
	L0	17.5167	0.7836
[0, 90°]	ATV	18.8412	0.8234
	FBPConvNet	20.2429	0.8152
	**SARTConvNet**	**22.6658**	**0.8527**
	L0	21.2196	0.8506
[0, 120°]	ATV	22.4382	0.8828
	FBPConvNet	23.9244	0.8822
	**SARTConvNet**	**25.1403**	**0.9077**
	L0	25.0614	0.9004
[0, 150°]	ATV	26.2771	0.9354
	FBPConvNet	27.7295	0.9239
	**SARTConvNet**	**29.7526**	**0.9456**

**Table 5:** Quantitative results associated with different algorithms for the chest image from different angle noise-add projection data.

Scanning ranges	Algorithm	PSNR	SSIM
	L0	16.6079	0.8087
[0, 90°]	ATV	17.1182	0.8220
	FBPConvNet	16.9948	0.8225
	**SARTConvNet**	**20.9816**	**0.8788**
	L0	21.5694	0.8980
[0, 120°]	ATV	22.1944	0.9041
	FBPConvNet	22.9651	0.8876
	**SARTConvNet**	**23.8609**	**0.9282**
	L0	25.4256	0.9490
[0, 150°]	ATV	26.1960	0.9530
	FBPConvNet	27.1494	0.9372
	**SARTConvNet**	**27.3750**	**0.9593**

## Discussion

### Training loss

**Fig 14:**
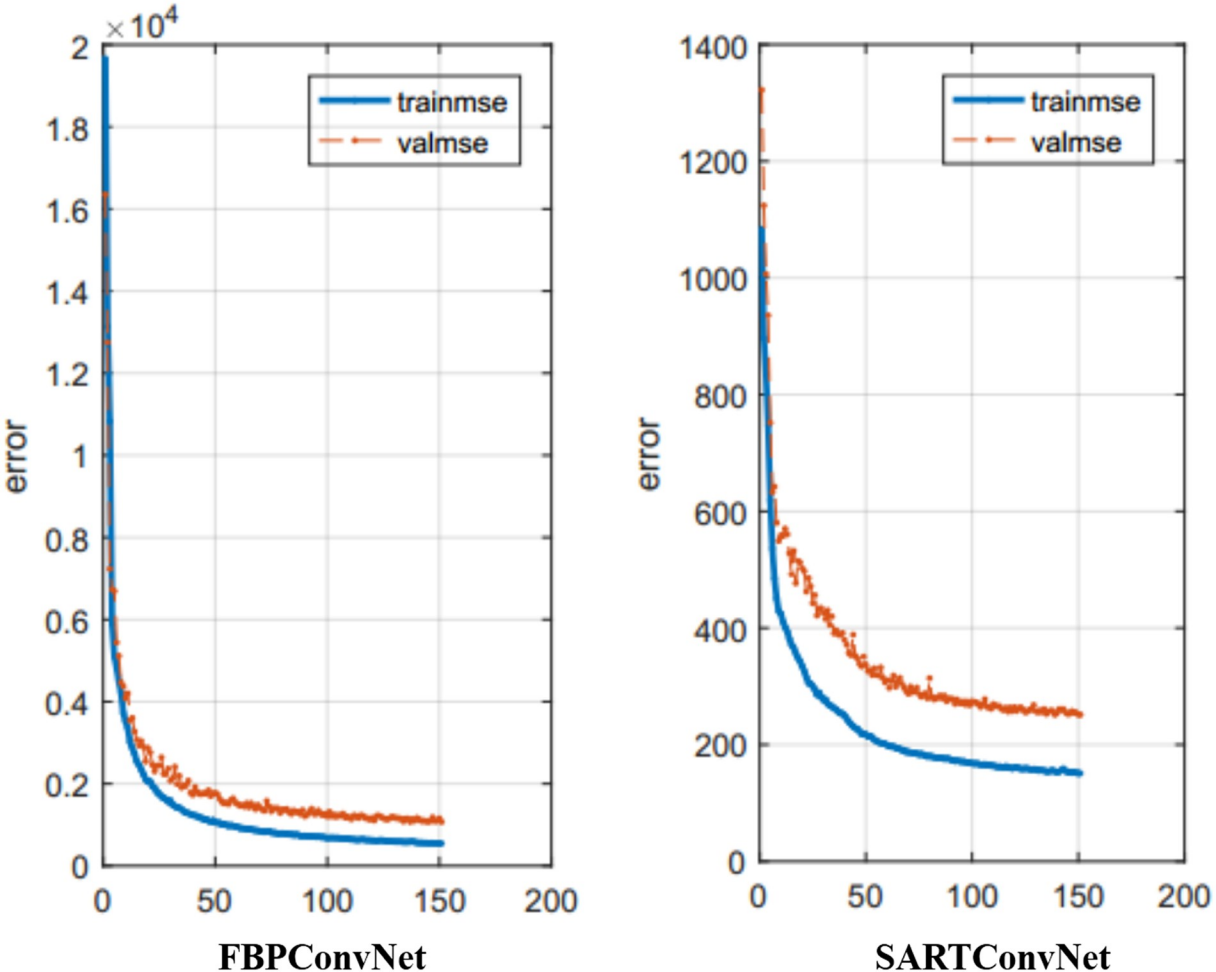
Loss function value changes in CNN training changes with epochs for both training dataset and testing dataset.

The training process of the CNN might be caught in the well-known over-fitting problem, and this U-net had used a technique named skip connection, which is added between the input and the output, to alleviate this problem. Then, we use the results from the experiment of the abdomen image for the scan range [0°, 150°] as an example to show how the loss function value changes with epochs for both the training dataset and the testing dataset (Fig 14). As is shown in Fig 14, the loss function value decreases with the increase of the number of epochs, and finally reaches a steady stage, which means the over-fitting problem is reduced to the lightest. In addition, the curve of the loss function value for the testing dataset has some oscillations at the beginning but will level off in the later stages.

### Execution time

We present the execution time of different algorithms in the experiment of the abdomen image for the scan ranges for the scan range [0°, 150°]. As shown in Table 6, the fast speed, which is indeed an advantage of deep learning, will disappear because we use the image reconstructed by SART method as the input image of the CNN. However, our method has two other advantages, one is that we do not need to choose the regularization term and adjust the regularization parameters, another one is that it produces better results than some state-of-the-art regularized iterative reconstruction methods. In addition, SART method, which is a fast algorithm among iterative reconstruction algorithms, can also be accelerated by the computer hardware and CUDA technique.

**Table 6:** Execution time with different algorithms.

Algorithms	Time (s)
L0	513
ATV	960
FBPConvNet	13
SARTConvNet	85

## Conclusions and perspectives

As a low-end CT system, TCT is in urgent demand in developing countries. To reduce scan time, decrease X-ray radiation or scan some long objects, furthermore, to avoid the inconsistency of the detector for the large angle scanning in translational scanning scheme, we use a limited-angle TCT scanning mode which introduces some additional noise and artifacts that seriously degrade the imaging quality and affect the accuracy, due to it is short of the continuous angular projection data. In this study, we develop a deep learning based limited-angle TCT image reconstruction algorithm. Experimental results show that this proposed method using the SART method is better than using the FBP method in the limited-angle TCT scanning mode, and the proposed method also has an excellent performance on suppressing the noise and the limited-angle artifacts while preserving the image structures.

The new algorithm improves the quality of the reconstructed image for limited-angle TCT scanning model, and it will be helpful for diagnosis. The main problem of our algorithm is that it needs a large dataset for training and an efficient computer is necessary. In the future, we can improve the generalization ability of our algorithm and extend it to higher dimensional cases such as 3D reconstruction to utilize more useful information. Although our algorithm is proposed for the limited-angle TCT, since the SART method and deep learning technique will not be influenced by the geometrical structure of the scanning mode, our algorithm can be extended to the generic limited angle tomography, such as C-arm cone-beam CT.

In conclusion, this paper uses a limited-angle TCT scanning model and develops a deep learning based limited-angle TCT image reconstruction algorithm. Some databases are used to evaluate the performance of the proposed method in comparison with other three methods. The experimental results demonstrate that our algorithm exhibits better performance in terms of suppressing the noise and the limited-angle artifacts while preserving the image structures.
